# Privacy-Preserving Distributed Analytics in Fog-Enabled IoT Systems

**DOI:** 10.3390/s20216153

**Published:** 2020-10-29

**Authors:** Liang Zhao

**Affiliations:** Department of Information Technology, Kennesaw State University, Marietta, GA 30060, USA; lzhao10@kennesaw.edu

**Keywords:** privacy-preserving, distributed analytics, fog computing, internet of things

## Abstract

The Internet of Things (IoT) has evolved significantly with advances in gathering data that can be extracted to provide knowledge and facilitate decision-making processes. Currently, IoT data analytics encountered challenges such as growing data volumes collected by IoT devices and fast response requirements for time-sensitive applications in which traditional Cloud-based solution is unable to meet due to bandwidth and high latency limitations. In this paper, we develop a distributed analytics framework for fog-enabled IoT systems aiming to avoid raw data movement and reduce latency. The distributed framework leverages the computational capacities of all the participants such as edge devices and fog nodes and allows them to obtain the global optimal solution locally. To further enhance the privacy of data holders in the system, a privacy-preserving protocol is proposed using cryptographic schemes. Security analysis was conducted and it verified that exact private information about any edge device’s raw data would not be inferred by an honest-but-curious neighbor in the proposed secure protocol. In addition, the accuracy of solution is unaffected in the secure protocol comparing to the proposed distributed algorithm without encryption. We further conducted experiments on three case studies: seismic imaging, diabetes progression prediction, and Enron email classification. On seismic imaging problem, the proposed algorithm can be up to one order of magnitude faster than the benchmarks in reaching the optimal solution. The evaluation results validate the effectiveness of the proposed methodology and demonstrate its potential to be a promising solution for data analytics in fog-enabled IoT systems.

## 1. Introduction

The Internet of Things (IoT) is a system of interconnected devices and networks in which information can be gathered from the surrounding environment. With the large deployment of IoT-based systems, the data volume collected is expanding significantly [1]. However, the data generated from IoT devices would be utilized and turned into insights only if it goes through analysis. While traditional Cloud-based solution enjoys the simple architecture by collecting all the raw data into a central place for processing and analytics, it is not suitable for many time-sensitive applications due to its limitations as follows. (1) Data moving is costly and sometimes it is even infeasible to transfer all the IoT raw data to Cloud for analytics due to bandwidth and limitation. (2) The latency is high considering the communication between edge devices and Cloud, which prevents fast response in latency critical IoT applications.

**Figure 1:**
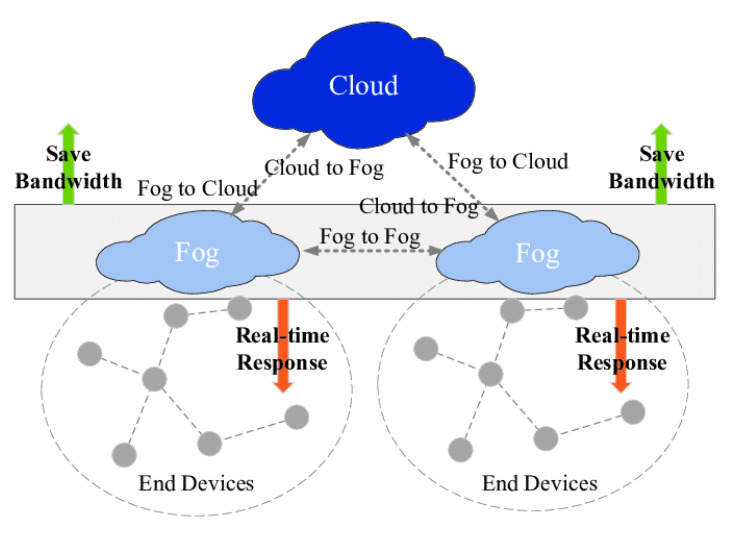
Fog-enabled IoT system infrastructure [7].

Fog computing is emerging as an alternative to the traditional Cloud-based solution by pushing processing and analytics near to where the data are generated [2]. It adds another middle fog layer between IoT devices and Cloud containing fog nodes that are placed close to edge devices (see Figure 1). The fog enables computation to be closer to the things that produce and act on IoT data. Fog nodes are usually considered to carry out a substantial amount of computation, storage, and communication in proximity to IoT edge devices in order to reduce the latency. It avoids the possible long-distance communication between edge devices to Cloud and mitigates heavy computation and analytics on Cloud. Although this architecture is promising in reducing latency by offloading data or computation tasks from edge devices into fog nodes [3,4], a careful design is demanded for efficient implementation of analytics. Distributed algorithms can play an important role in this context in which the computational capacities of all the participants in the system are leveraged to enable more robust and optimized solutions for data analytics. The key challenge lies on balancing the computation and communication for the all the IoT devices and fog nodes in the IoT system.

Data analytics is powerful in facilitating decision-making process while privacy concerns have also arisen in many data related applications. In distributed solutions for data analytics, they require information exchange among edge devices and fog nodes. In some cases, although the raw data are not transferred, the private information about their raw data can be obtained from their computed quantity such as gradients or model parameters as these are computed based on its local data [5,6]. To address this concern, a privacy-preserving data analytics scheme becomes imperative for adopting data-driven solutions in various fields in practice.

The main contribution of this paper is three-fold: (1) We design a new distributed data analytics algorithm for fog-enabled IoT systems. (2) We further develop a privacy-preserving secure protocol based on the proposed distributed algorithm with homomorphic encryption. (3) We evaluate our proposed protocol on two case studies: seismic imaging and diabetes progression prediction. The experiment results demonstrate that the proposed secure protocol not only achieves data privacy but also outperforms the benchmarks in terms of convergence speed. We believe this is an important addition to existing infrastructures for efficient and secure data analytics in fog-enabled IoT systems.

The organization of this paper is as follows. Section 2 discusses the related work in distributed data analytics and privacy-preserving schemes. Section 3 describes the design of the proposed distributed algorithm. Section 4 presents the secure privacy-preserving protocol based on the distributed algorithm. Section 5 demonstrates the evaluation of the proposed protocol on three applications. Finally, Section 6 concludes this paper with future directions.

## 2. Related Work

### 2.1. Distributed Analytics

Distributed computing has attracted much attention recently in various communities. In signal processing community, a line of research is focusing on developing distributed algorithm for state estimation in sensor networks as the number of sensors is increasing significantly aiming to provide a better service. A distributed algorithm for least-squares estimation is proposed in [8] leveraging the space-time structure of the data. A series of work on solving recursive least-square in a distributed manner is proposed in [9,10,11,12]. These works are based on the diffusion strategy that all the nodes in the network cooperate with each other in order to spread the information across the entire network. Mateos and Giannakis studied the stability and performance of various distributed recursive leas-square algorithms [13]. A gossip based distributed algorithm is proposed in [14] considering more general signal processing problems. In the aforementioned works, a fusion center is needed in the network for processing the local information gathered and is responsible for sending the mixed estimates back to the nodes.

In the numerical optimization community, many efforts have been made to develop distributed optimization algorithms in recent years due to the demand in applications such as multi-agent control and distributed machine learning in which data analytics problems can be modeled as convex optimization. In particular, fully distributed solutions are considered in the literature that all the nodes in the network collaborate with each other by exchanging information with their immediate neighbors only. The goal is that all the nodes can obtain the same optimal solution as in the centralized setting. In the fully distributed setting, algorithms can be categorized into synchronous or asynchronous depending on the behavior of local nodes for communicating with others. Gradient-type distributed algorithms for solving convex optimization are proposed in [15,16,17,18,19]. These works are based on a synchronous model such that each node needs to wait for its slowest neighbor to proceed. In addition, it has been found that the exact optimal solution cannot be achieved with fixed step sizes [20]. On the other hand, diminishing step sizes are adopted to guarantee the convergence to the optimal solution in [19,21,22,23,24,25,26,27]. However, the resulting convergence speed is relatively slower than its counterpart with fixed step sizes. The method proposed in [28] was proved to have an $O(1/k^{2})$ rate in terms of objective value error over the iteration number *k*. This convergence rate matches with the optimal rate for general gradient-based methods in centralized setting while iteration dependent consensus communication is required in each iteration causing the degraded performance in practice. To address this issue, a new algorithm is proposed in [29] allowing convergence to the exact solution using fixed step sizes. In contrast to the aforementioned synchronous distributed algorithms, several asynchronous solutions have been developed in the literature such that each node can perform its action independent of other nodes [23,30,31]. The methods in [30,31] combine the computation scheme of alternating direction method of multipliers (admm) and random gossip for communication [32]. An asynchronous model for distributed optimization is developed in [23], in which the global variable is split among the nodes and each node is responsible for a partial of it. A random broadcast based asynchronous algorithm is designed in [33], which adopts a gradient-based computation scheme for local nodes. A modified algorithm has been proposed by replacing gradient step with full optimization in order to speed up the convergence speed and thus reduce the communication cost [34].

### 2.2. Privacy-Preserving Schemes

Many privacy-preserving schemes have been proposed for data analytics in the literature. Differential privacy is a new approach tailored to the problem of privacy-preserving data analytics such that individuals or organizations can leverage sensitive data for better service without privacy breach [35,36]. It has a wide range of applications in many areas, such as recommender system [37] and genomics [38]. The foundation of differential privacy is to perturb the query output by adding random noise and many studies have focused on designing better noise-adding mechanisms [39]. A fundamental trade-off exists in differential privacy that the accuracy of the solution depends on the level of the noise added.

Another line of privacy-preserving schemes resorts to cryptographic-based techniques in which private data would be encrypted before analytics [40]. Homomorphic encryption is a type of encryption allowing one to directly operate on encrypted data [41]. It can be applied in data analytics for privacy-centric industries such as healthcare in which raw data cannot be leaked to any entity except the data holder. Several partially homomorphic cryptosystems have been proposed since 1980s allowing specific and limited operations on encrypted data. For instance, Goldwasser—Micali cryptosystem allows exclusive or operation [42], and Paillier cryptosystem allows unbounded number of modular additions [43]. More recently, several generations of fully homomorphic cryptosystems have been designed, which support arbitrary computation on encrypted data. Although fully homomorphic cryptosystems have great potentials in outsourcing private computations, they are relatively more time-consuming compared to their partially homomorphic counterparts.

## 3. Distributed Algorithm Design

In this section, we first discuss the formulation for data analytics problems and then present our proposed distributed algorithm. Many data analytics problems such as least-squares, logistic regression, and support vector machines can be formulated as convex optimization problems as follows with objective function F(x) to be convex [44].

(1) $$\min_{x\in X}F\left(x\right).$$

In practice, iterative methods are usually adopted for solving Equation (1), and, among them, first-order methods such as gradient based algorithms are very popular in particular for big data analytics since it just requires the gradient information, which is relatively cheap to compute comparing to second-order methods such as Newton’s method, which needs to compute the hessian [44]. In this paper, our focus is thus on gradient methods and our proposed distributed algorithm relies on a decomposed formulation of Equation (1) fitting into the infrastructure of fog-enabled IoT system that computational resources are located in a distributed manner.

### 3.1. Decomposed Problem Formulation

We discuss the decomposed problem formulation for Equation (1) in this subsection. Consider that there are *m* data generation places in the system and each place *i* has a local private objective function $F_{i}:R^{n}\to R$ characterized by its data acquired and the analytics model. The resulting optimization problem can be expressed as follows.

(2) $$\min_{x\in X}\left\{F\left(x\right):=\sum_{i=1}^{m}F_{i}\left(x\right)\right\}.$$

It can be seen that the global objective function is the summation of all the local private objective functions. Now, assume there are *p* main computation nodes in the system and each computation node corresponds to a subset of the *m* local objective functions without overlap. Note that, if the number of computation nodes is the same as the number of data generation places such that p=m, it means each computation node *i* can access a local private function $F_{i},i\in \left\{1,2,\ldots ,m\right\}$. The goal is that all the computation nodes can obtain the optimal solution x∈X minimizing Equation (2) by evaluating the local objective functions they can access to and exchanging information with each other.

The formulation aforementioned has connections to multi-agent control, distributed signal processing and statistical learning problems investigated in various communities [45,46,47]. In the literature, it has been used in various applications such as sensor networks, smart manufacturing, and power systems [48,49].

### 3.2. Distributed Algorithm

In this section, we describe our distributed algorithm for solving Equation (2) in the fog computing enabled IoT system architecture (see Figure 1). The proposed algorithm consists of two parts: the procedure for fog nodes and edge devices, respectively. Assume that there are *m* edge devices in the system and they hold the local data generated. Note that each edge device *i* has a private local objective function $F_{i}$ corresponding to the term in Equation (2). The function $F_{i}$ is determined by the model/problem they want to solve and also device *i*’s local data. We assume that there are *p* fog nodes and each fog node is responsible for an area that a certain set of edge devices will communicate with due to proximity. The procedures for fog nodes and edge devices are described, respectively.

**Fog** **nodes:** In each iteration, a pair of fog nodes are selected to exchange their estimates. The mixed estimates would be sent to their corresponding edge devices and the fog nodes would wait for edge devices’ returned gradients for updating their estimates. All other fog nodes that have not been selected in the current iteration perform in a similar way but send their individual estimates to edge devices instead.
**Edge** **devices:** In each iteration, the edge devices compute the gradients with respect to the estimates (received from the fog nodes) using their local objective functions $F_{i}$ and return them back to the fog nodes for updating estimates.

Notice that fog nodes are the main computation points in this process, and they update and exchange the estimates with each other. Edge devices are the raw data holders and they send back only their calculated local gradients. In the whole process, raw data held by edge devices have not been moved anywhere and kept localized. The details of their procedures are summarized in Algorithms 1 and 2, respectively.

**Remark** **1.** The fog nodes’ estimates are designed to reach consensus eventually and thus we can pick any node’s estimate as the final solution. This final solution can be transmitted to Cloud for backup. For latency-sensitive applications where a solution is needed within a short period of time in which fog nodes’ solutions are not consensual yet, the final solution will be obtained by averaging all the estimates.

**Algorithm 1:** Fog node procedure**Input:** Starting point $x_{0}^{1}=x_{-1}^{1},x_{0}^{2}=x_{-1}^{2},\cdots ,x_{0}^{p}=x_{-1}^{p}$. Initialize the iteration number *k*. $\beta_{k}^{i}$ and $\eta_{k}^{i}$ are momentum and step size parameters used by fog node *i* at iteration *k*.1: **while** the stopping criterion have not been reached, all the fog nodes **do**2:  **if** fog node *i*’s clock ticks at iteration *k*, and selects a neighboring fog node *j*, **then**3:   Node *i* and *j* exchange their current estimates $x_{k-1}^{i}$ and $x_{k-1}^{j}$ and update in parallel.4:   Fog node *i* updates as follows.5:   $y_{k}^{i}=\frac{1}{2}\left(x_{k-1}^{i}+x_{k-1}^{j}\right)+\beta_{k}^{i}\left(x_{k-1}^{i}-x_{k-2}^{i}\right)$,6:   Fog node *i* sends mixed estimate $y_{k}^{i}$ to its corresponding edge devices.7:   Fog node *i* waits for the edge devices to return their gradients and aggregate them (the summation) as $g_{k}^{i}$.8:   Fog node *i* updates its estimate $x_{k}^{i}=y_{k}^{i}-\eta_{k}^{i}g_{k}^{i}$.9:   Fog node *j* updates as follows.10:   $y_{k}^{j}=\frac{1}{2}\left(x_{k-1}^{j}+x_{k-1}^{i}\right)+\beta_{k}^{j}\left(x_{k-1}^{j}-x_{k-2}^{j}\right)$,11:   Fog node *j* sends mixed estimate $y_{k}^{j}$ to its corresponding edge devices.12:   Fog node *j* waits for the edge devices to return their gradients and aggregate them (the summation) as $g_{k}^{j}$.13:   Fog node *j* updates its estimate $x_{k}^{j}=y_{k}^{j}-\eta_{k}^{j}g_{k}^{j}$.14:   Other fog nodes *q*, which are not *i* or *j* update as follows.15:   $y_{k}^{q}=x_{k-1}^{q}+\beta_{k}^{q}\left(x_{k-1}^{q}-x_{k-2}^{q}\right)$,16:   Fog node *q* sends mixed estimate $y_{k}^{q}$ to its corresponding edge devices.17:   Fog node *q* waits for the edge devices to return their gradients and aggregate them (the summation) as $g_{k}^{q}$.18:   Fog node *q* updates its estimate $x_{k}^{q}=y_{k}^{q}-\eta_{k}^{q}g_{k}^{q}$.19:  **end if**20:  Increment *k*.21: **end while**22: Send EXIT signal.
**Algorithm 2:** Edge device procedure1: **while** EXIT signal has not been received, each edge device *j* with *j* belongs to the set of edge devices that associated with fog node *i*
**do**2:   Edge device *j* receives edge node *i*’s mixed estimate $y_{k}^{i}$.3:   Edge device *j* computes the gradient with respect to $y_{k}^{i}$ using its local objective function $F_{j}$.4:   Edge device *j* sends the computed gradient to its corresponding fog node *i*.5: **end while**


**Complexity Analysis for Algorithms 1 and 2:** Assume there are *m* edge devices and *p* fog nodes in the system. In each iteration, there are O(m) gradient evaluations performed by *m* edge devices in parallel. There are O(p) local updates conducted by *p* fog nodes in parallel. The calculations involved in fog nodes’ updates are mainly vector addition and subtraction. For communication, there are O(2m) communications between edge devices and their fog nodes in each iteration. There are O(2) communications between fog nodes since only a pair of fog nodes is selected in each iteration for exchanging their estimates. The size of each communication is same as the size of the decision vector. Notice that the communication complexity is constant with respect to the number of fog nodes *p*.

### 3.3. Algorithm Interpretation

In this subsection, we show the rationale of proposing Algorithms 1 and 2 in solving (2). To solve the centralized problem in (1), a well-known first-order iterative method is gradient descent and the update rule can be described as follows.

(3) $$x_{k}=x_{k-1}-\eta \nabla F\left(x_{k-1}\right),$$

where $x_{k}$ is the estimate for the solution at iteration *k*, η is the step size parameter and $\nabla F\left(x_{k-1}\right)$ is the gradient. In [50,51], Nesertov developed an accelerated gradient descent, which speeds up the convergence using a multi-step strategy as follows.

(4) $$\begin{array}{cc} y_{k} & =x_{k-1}+\beta_{k}\left(x_{k-1}-x_{k-2}\right),\\ x_{k} & =y_{k}-\eta_{k}\nabla F\left(y_{k}\right), \end{array}$$

where $y_{k}$ is an auxiliary variable, $\beta_{k}\left(x_{k-1}-x_{k-2}\right)$ is called the “momentum” term, and $\beta_{k}$ is the momentum parameter. Nesterov proved the optimality of his proposed method in the sense that it achieves the best convergence rate assuming only function value and gradient information is available.

In the proposed distributed algorithm, we adopted the Nesterov’s accelerated gradient method in (4) as the update rule for the fog nodes (see Steps 15–18 in Algorithm 1). However, it is clearly suboptimal if all the fog nodes only update their estimates since they only have partial knowledge about the system without communicating with other fog nodes. Hence, the other part is designing communication scheme for fog nodes to exchange information with each other in order to fuse their estimates such that all the nodes can reach the optimal solution for the global problem. To minimize the communication overhead, we adopted the random gossip scheme developed in [32] that in each iteration only a pair of nodes exchange and take the average of their estimates as the new estimate. Although the original problem investigated in [32] is average consensus, it can be extended to more general optimization problems. In addition, it is the key to guarantee consensus of all the computation nodes in the distributed setting. The difference in our proposed algorithm is that we allow other nodes that are not selected in the current iteration to update locally. This setting is due to the trade-off between communication and computation in distributed networks [52] such that, if local nodes work harder, it could potentially reduce the communication rounds among the computation nodes towards convergence. Thus, our update rule for fog nodes combining Nesterov’s gradient method and random gossip communication is as follows (for node *i*):

(5) $$\begin{array}{cc} y_{k}^{i} & =\frac{1}{2}\left(x_{k-1}^{i}+x_{k-1}^{j}\right)+\beta_{k}^{i}\left(x_{k-1}^{i}-x_{k-2}^{i}\right),\\ x_{k}^{i} & =y_{k}^{i}-\eta_{k}^{i}g_{k}^{i}, \end{array}$$

where we assume fog nodes *i* and *j* are selected at iteration *k* and exchange their estimates. $g_{k}^{i}$ is the aggregated gradient from edge devices in fog area *i*. Notice that our proposed distributed algorithm is a mimic of Nesterov’s optimal accelerated gradient method in the centralized setting.

### 3.4. An Illustrative Example of Executing the Distributed Algorithm

**Figure 2:**
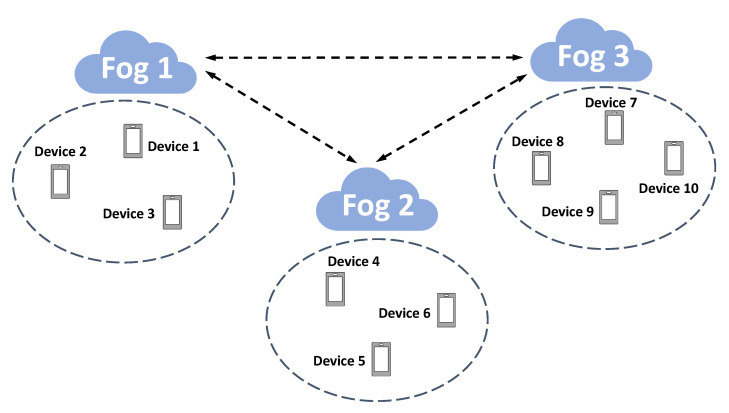
An illustrative example of executing Algorithms 1 and 2.

In this subsection, we discuss a concrete example of executing the proposed distributed algorithms in a fog-enabled IoT system.The architecture is illustrated in Figure 2. There are three fog nodes in the system. In Fog Area 1, there are Edge Devices 1–3, Fog Area 2 has Edge Devices 4–6, and Fog Area 3 contains Edge Devices 7–10. Let $N_{i}$ be the set of neighbors for fog node *i*; then, it can be seen that $N_{1}=\left\{2,3\right\}$, $N_{2}=\left\{1,3\right\}$, $N_{3}=\left\{1,2\right\}$. Algorithms 1 and 2 run as follows.

Iteration 1: Fog node 2’s clock ticks and it selects node 1 for exchanging their estimates $x_{0}^{2}$ and $x_{0}^{1}$. Fog node 2 computes $y_{1}^{2}=\frac{1}{2}\left(x_{0}^{2}+x_{0}^{1}\right)+\beta_{1}^{2}\left(x_{0}^{2}-x_{-1}^{2}\right)$ and then node 2 sends mixed estimate $y_{1}^{2}$ to its corresponding Edge Devices 4-6. Edge Devices 4–6 compute their gradients using their private functions with respect to $y_{1}^{2}$. These gradients $\nabla F_{4}\left(y_{1}^{2}\right),\nabla F_{5}\left(y_{1}^{2}\right),\nabla F_{6}\left(y_{1}^{2}\right)$ are returned to fog node 2 and aggregated as $g_{1}^{2}\leftarrow \nabla F_{4}\left(y_{1}^{2}\right)+\nabla F_{5}\left(y_{1}^{2}\right)+\nabla F_{6}\left(y_{1}^{2}\right)$. Fog node 2 updates its estimate $x_{1}^{2}=y_{1}^{2}-\eta_{1}^{2}g_{1}^{2}$.
  For fog node 1, it computes $y_{1}^{1}=\frac{1}{2}\left(x_{0}^{1}+x_{0}^{2}\right)+\beta_{1}^{1}\left(x_{0}^{1}-x_{-1}^{1}\right)$ and then sends mixed estimate $y_{1}^{1}$ to its corresponding Edge Devices 1–3. Edge Devices 1–3 compute their gradients using their private functions with respect to $y_{1}^{1}$. These gradients $\nabla F_{1}\left(y_{1}^{1}\right),\nabla F_{2}\left(y_{1}^{1}\right),\nabla F_{3}\left(y_{1}^{1}\right)$ are returned to fog node 1 and aggregated as $g_{1}^{1}\leftarrow \nabla F_{1}\left(y_{1}^{1}\right)+\nabla F_{2}\left(y_{1}^{1}\right)+\nabla F_{3}\left(y_{1}^{1}\right)$. Fog node 1 updates its estimate $x_{1}^{1}=y_{1}^{1}-\eta_{1}^{1}g_{1}^{1}$.
  The remaining fog node 3 receives signal that it will not exchange its estimate with others and thus update as follows. It calculates $y_{1}^{3}=x_{0}^{3}+\beta_{1}^{3}\left(x_{0}^{3}-x_{-1}^{3}\right)$ and then sends mixed estimate $y_{1}^{3}$ to its corresponding Edge Devices 7–10. Edge Devices 7–10 compute their gradients using their private functions with respect to $y_{1}^{3}$. These gradients $\nabla F_{7}\left(y_{1}^{3}\right),\nabla F_{8}\left(y_{1}^{3}\right),\nabla F_{9}\left(y_{1}^{3}\right),\nabla F_{1}0\left(y_{1}^{3}\right)$ are returned to fog node 3 and aggregated as $g_{1}^{3}\leftarrow \nabla F_{7}\left(y_{1}^{3}\right)+\nabla F_{8}\left(y_{1}^{3}\right)+\nabla F_{9}\left(y_{1}^{3}\right)+\nabla F_{1}0\left(y_{1}^{3}\right)$. Fog node 3 updates its estimate $x_{1}^{3}=y_{1}^{3}-\eta_{1}^{3}g_{1}^{3}$.

In the next iteration, the scheme will be executed in the same fashion as in Iteration 1 until the stopping criteria have been reached. Notice that in Algorithms 1 and 2, the raw data gathered by edge devices remain localized and edge nodes’ gradient information and fog nodes’ estimates are communicated. In the next section, we introduce a secure protocol further enhancing the privacy of each participant in the computation and analytics processes.

## 4. Secure Privacy-Preserving Protocol

In this section, we present a secure protocol further enhancing the privacy of each participant in the computation and analytics processes. As a motivating example, we show below that fog nodes can invert the gradients received from edge devices and obtain their raw data.

**Example:**  In Algorithm 2, edge devices computes their gradients and return them to their corresponding fog nodes. Assume that edge device *j* computes the gradient with respect to $y_{k}^{i}$ and sends it to fog node *i*. In addition, assume that the model we use is least square such that the global objective is as follows.
  (6) $$\min_{x}\;\;\frac{1}{2}{∥Ax-b∥}_{2}^{2}.$$
  Following the decomposed formulation in Section 3.1, the local objective function for edge device *j* can be expressed as follows.
  (7) $$F_{j}=\frac{1}{2}{∥A_{j}x-b_{j}∥}_{2}^{2},$$
  where matrix $A_{j}$ and vector contain the raw data for device *j*. The gradient of $F_{j}$ is:
  (8) $$\nabla F_{j}\left(x\right)=\frac{1}{2}\left(A_{j}^{T}A_{j}x-A_{j}^{T}b_{j}\right),$$
  where $A_{j}^{T}$ represents the transpose of matrix $A_{j}$. Assume that fog node *i* keeps the received edge device *j*’s gradient at iteration *k* and k+1 and they are:
  (9) $$\begin{array}{c} \nabla F_{j}\left(y_{k}^{i}\right)=\frac{1}{2}\left(A_{j}^{T}A_{j}y_{k}^{i}-A_{j}^{T}b_{j}\right),\\ \nabla F_{j}\left(y_{k+1}^{i}\right)=\frac{1}{2}\left(A_{j}^{T}A_{j}y_{k+1}^{i}-A_{j}^{T}b_{j}\right), \end{array}$$
  Taking the difference between the two equations in (9) yields: $\nabla F_{j}\left(y_{k}^{i}\right)-\nabla F_{j}\left(y_{k+1}^{i}\right)=\frac{1}{2}A_{j}^{T}A_{j}\left(y_{k}^{i}-y_{k+1}^{i}\right)$. If fog node *i* knows that edge device is using least square model, it can obtain $A_{j}^{T}A_{j}$ and then $A_{j}$ accordingly. Putting $A_{j}$ into any of the two equations in (9) yields $b_{j}$. At this point, edge device’s raw data has been leaked to fog node *i*.

In Algorithms 1 and 2, there are three types of communication involved: edge device to edge device, edge device to its corresponding fog node, and fog node to fog node. Hence, our goal is to protect each entity’s exact private information so that it will not be leaked during the aforementioned interactions. Our proposed secure protocol is based on the Pailier cryptosystem [43], which belongs to the category of homomorphic encryption schemes [41] allowing one to directly perform computations on encrypted data. The background of Pailier encryption is introduced first and then the design of the secure privacy-preserving protocol is discussed.

### 4.1. Paillier Cryptosystem

In this subsection, we briefly introduce the basics of Paillier cryptosystem. The scheme works as follows [43].

**Key generation:**

1. Select two equal length large prime numbers *p* and *q*.
2. Calculate n=pq and set g=n+1.
3. Set λ=ϕ(n) where $\phi \left(n\right)=\left(p-1\right)\left(q-1\right)$ is Euler’s totient function.
4. Find $\mu =\phi {\left(n\right)}^{-1}\;\mathrm{mod}\;n$ and $\phi {\left(n\right)}^{-1}$ is the modular multiplicative inverse of ϕ(n).
5. The public (encryption) key: $\left(n,g\right)$.
6. The private (decryption) key: $\left(\lambda ,\mu \right)$.

**Encryption:**

1. Suppose *m* is the plaintext, where 0≤m<n. Select a random *r* where 0<r<n.
2. Calculate ciphertext as: $c=g^{m}\cdot r^{n}{\;\mathrm{mod}\;n}^{2}$.

**Decryption:**

1. Suppose *c* is the ciphertext, where 0≤m<n. Select a random *r* where 0<r<n.
2. Calculate the plaintext as: $m=L(c^{\lambda }{\;\mathrm{mod}\;n}^{2})\cdot \mu \;\mathrm{mod}\;n$, where $L\left(x\right)=\frac{x-1}{n}$.

**Homomorphic properties:**

1. The ciphertext of the sum of two messages can be obtained by the product of two individual ciphertexts of the messages, respectively.
2. Decrypting a ciphertext raised to a constant *k* yields the product of the plaintext and the constant.

### 4.2. Secure Protocol Design

**Figure 3:**
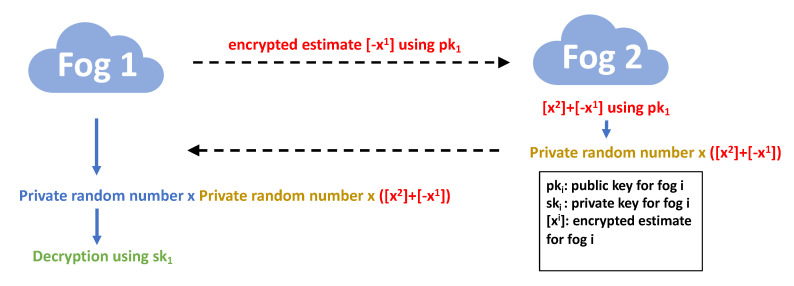
An example of secure interaction for fog nodes in Algorithm 3.

**Figure 4:**
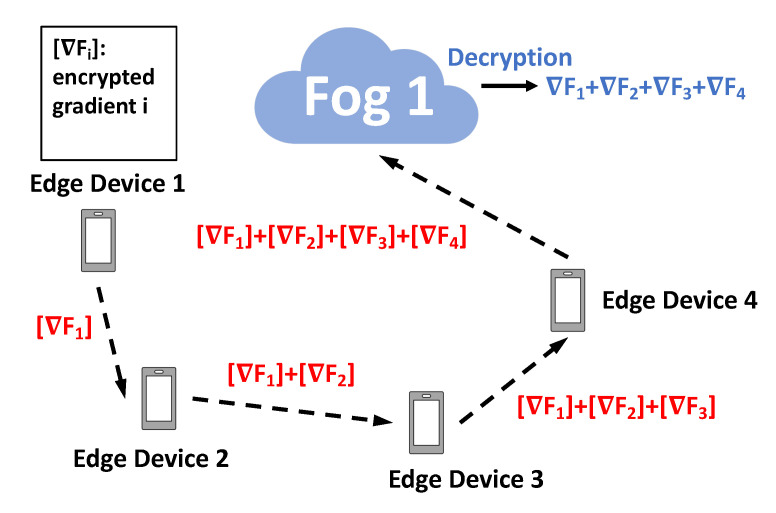
An example of secure interaction for edge devices in Algorithm 4.

The key challenge for designing the secure protocol lies on how to leverage the “addition” and “multiplication” homomorphic properties (shown at the end of Section 4.1) provided by Paillier encryption to perform the tasks in Algorithms 1 and 2. The details are described in Algorithms 3 and 4 for fog nodes and edge devices, respectively. To better illustrate the encryption based secure design, we show two examples of the interactions in the secure protocols. One is for secure exchange between two fog nodes used in Algorithm 3 (see Figure 3), while the other demonstrates the secure interaction between edge devices in Algorithm 4 (see Figure 4).

**Algorithm 3:** Secure fog node procedure**Input:** Starting point $x_{0}^{1}=x_{-1}^{1},x_{0}^{2}=x_{-1}^{2},\cdots ,x_{0}^{p}=x_{-1}^{p}$. Initialize the iteration number k=0. All the fog nodes generate their public and private key pairs. $\beta_{k}^{i}$ and $\eta_{k}^{i}$ are momentum and step size parameters used by fog node *i* at iteration *k*.1: **while** the stopping criterion have not been reached, all the fog nodes **do**2:  **if** fog node *i*’s clock ticks at iteration *k*, and selects a neighboring fog node *j*, **then**3:     Fog node *i* updates as follows.4:     Node *i* encrypts its estimate using its public key $pk_{i}$ and sends the encrypted estimate ${\left[-x_{k-1}^{i}\right]}_{pk_{i}}$ to node *j*.5:     Node *j* encrypts its own estimate using node *i*’s public key $pk_{i}$ and obtains ${\left[x_{k-1}^{j}\right]}_{pk_{i}}$. Perform the addition ${\left[x_{k-1}^{j}\right]}_{pk_{i}}+{\left[-x_{k-1}^{i}\right]}_{pk_{i}}$ and then multiply a private random number $\gamma_{ji}$ uniformly sampled from $\left[\sqrt{2}-1,1\right]$ to the summation and finally sends it back to node *i*.6:     Node *i* receives the message and decrypts it and then multiply with a private random number $\gamma_{ij}$ uniformly sampled from $\left[\sqrt{2}-1,1\right]$.7:     Node *i* obtain the mixed average as $x_{k-1}^{i}+\gamma_{ij}\times \gamma_{ji}\times \left(x_{k-1}^{j}-x_{k-1}^{i}\right)$.8:     $y_{k}^{i}=x_{k-1}^{i}+\gamma_{ij}\times \gamma_{ji}\times \left(x_{k-1}^{j}-x_{k-1}^{i}\right)+\beta_{k}^{i}\left(x_{k-1}^{i}-x_{k-2}^{i}\right)$,9:     Fog node *i* sends mixed estimate $y_{k}^{i}$ to its corresponding edge devices.10:   Fog node *i* waits for the summation of the encrypted gradients from the edge devices and then decrypts it as $g_{k}^{i}$ using its private key $sk_{i}$.11:   Fog node *i* updates its estimate $x_{k}^{i}=y_{k}^{i}-\eta_{k}^{i}g_{k}^{i}$.12:   Fog node *j* updates as follows.13:   Node *j* encrypts its estimate using its public key $pk_{j}$ and sends the encrypted estimate ${\left[-x_{k-1}^{j}\right]}_{pk_{j}}$ to node *i*.14:   Node *i* encrypts its own estimate using node *j*’s public key $pk_{j}$ and obtains ${\left[x_{k-1}^{i}\right]}_{pk_{j}}$. Perform the addition ${\left[x_{k-1}^{i}\right]}_{pk_{j}}+{\left[-x_{k-1}^{j}\right]}_{pk_{j}}$ and then multiply a private random number $\gamma_{ij}$ uniformly sampled from $\left[\sqrt{2}-1,1\right]$ to the summation and finally sends it back to node *j*.15:   Node *j* receives the message and decrypts it and then multiply with a private random number $\gamma_{ji}$ uniformly sampled from $\left[\sqrt{2}-1,1\right]$.16:   Node *j* obtain the mixed average as $x_{k-1}^{j}+\gamma_{ji}\times \gamma_{ij}\times \left(x_{k-1}^{i}-x_{k-1}^{j}\right)$.17:   $y_{k}^{j}=x_{k-1}^{j}+\gamma_{ji}\times \gamma_{ij}\times \left(x_{k-1}^{i}-x_{k-1}^{j}\right)+\beta_{k}^{j}\left(x_{k-1}^{j}-x_{k-2}^{j}\right)$,18:   Fog node *j* sends mixed estimate $y_{k}^{j}$ to its corresponding edge devices.19:   Fog node *j* waits for the summation of the encrypted gradients from the edge devices and then decrypts it as $g_{k}^{j}$ using its private key $sk_{j}$.20:   Fog node *j* updates its estimate $x_{k}^{j}=y_{k}^{j}-\eta_{k}^{j}g_{k}^{j}$.21:   Other fog nodes *q*, which are not *i* or *j* update as follows.22:   $y_{k}^{q}=x_{k-1}^{q}+\beta_{k}^{q}\left(x_{k-1}^{q}-x_{k-2}^{q}\right)$,23:   Fog node *q* sends mixed estimate $y_{k}^{q}$ to its corresponding edge devices.24:   Fog node *q* waits for the summation of the encrypted gradients from the edge devices and then decrypts it as $g_{k}^{q}$ using its private key $sk_{q}$.25:   Fog node *q* updates its estimate $x_{k}^{q}=y_{k}^{q}-\eta_{k}^{q}g_{k}^{q}$.26:  **end if**27:  Increment *k*.28: **end while**29: Send EXIT signal.

**Algorithm 4:** Secure edge device procedure1: **while** EXIT signal has not been received, each edge device *j* with *j* belongs to the set of edge devices that associated with fog node *i*
**do**2:   Edge device *j* receives fog node *i*’s mixed estimate $y_{k}^{i}$.3:   Edge device *j* computes the gradient with respect to $y_{k}^{i}$ using its local objective function $F_{j}$.4:   Edge device *j* encrypts its gradient using its corresponding fog node *i*’s public key $pk_{i}$.5:   The edge devices belong to the area of fog node *i* pass and do summation on their encrypted gradient in order.6:   The last edge device with the summation of all the gradients sends the aggregated encrypted gradients to its corresponding fog node *i*.7: **end while**

In the following theorem, we verify the “correctness” of the secure protocol in the sense that the solution obtained from the secure protocol (Algorithms 3 and 4) is approximately the same as its counterpart in Algorithms 1 and 2 without encryption.

**Theorem** **1.** *In expectation, each fog node i’s estimate $x_{k}^{i}(\;i=1,2,\cdots ,p$) at iteration k obtained from executing the secure protocols in Algorithms 3 and 4 is the same as the counterpart obtained from the distributed algorithms in Algorithms 1 and 2.*

**Proof.**  First, we discuss the similarity between Algorithms 2 and 4. Algorithm 4 involves the sum of encrypted gradients and the decrypted message obtained by fog *i* would be the same as the aggregated gradients in Algorithm 2 according to the “addition” homomorphic property (shown in the first property at the end of Section 4.1). Next, for Algorithm 3, after Step 6, fog node *i* obtains $\gamma_{ij}\times \gamma_{ji}\times \left(x_{k-1}^{j}-x_{k-1}^{i}\right)$. Since $\gamma_{ij},\gamma_{ji}∼U\left(\sqrt{2}-1,1\right)$ are two independent random variables uniformly sampled, the expectation of $\gamma_{ij}\times \gamma_{ji}\times \left(x_{k-1}^{j}-x_{k-1}^{i}\right)$ is $E\left(\gamma_{ij}\times \gamma_{ji}\times \left(x_{k-1}^{j}-x_{k-1}^{i}\right)\right)=\frac{1}{2}\left(x_{k-1}^{j}-x_{k-1}^{i}\right)$ and thus the expectation of the value in Step 7 is $E\left(x_{k-1}^{i}+\gamma_{ij}\times \gamma_{ji}\times \left(x_{k-1}^{j}-x_{k-1}^{i}\right)\right)=\frac{1}{2}\left(x_{k-1}^{j}+x_{k-1}^{i}\right)$, which is the same as the counterpart in Step 5 of Algorithm 1. This same reasoning can be applied to fog node *j* and the remaining follows. This completes the proof for Theorem 1. □

**Complexity analysis for Algorithms 3 and 4:** Assume there are *m* edge devices and *p* fog nodes in the system. In each iteration, there are O(m) gradient evaluations performed by *m* edge devices in parallel. There are O(p) local updates conducted by *p* fog nodes in parallel. The calculations involved in fog nodes’ updates are mainly vector addition and subtraction except the two fog nodes selected for exchanging encrypted information in each iteration. For communication, there are O(m−1) communications between edge devices in each iteration. There are O(m+1) communications between fog nodes and their edge devices. There are O(4) communications between fog nodes since only a pair of fog nodes is chosen for communication in each iteration. The size of each communication is same as the size of the decision vector. Notice that the communication complexity is constant with respective to the number of fog nodes *p*.

### 4.3. Security Analysis

In this subsection, we analyze the security of our proposed protocols described in Algorithms 3 and 4, respectively. There are three types of communication involved in the proposed protocols: between fog nodes, between edge devices, and between fog nodes and edge devices. Our security goal is that exact private information cannot be obtained by their neighbors who communicate with them. Note that this goal is referred to as privacy-preserving computation or secure multi-party computation in the literature, and it is different from the conventional security goals aiming to prevent information leak from outsiders during communication [53,54,55]. The analysis is summarized in the following theorems and the associated proofs demonstrate that our invented secure protocols are capable of protecting participants’ privacy from each other during the entire computation and data analytics process.

**Theorem** **2.** *Assume all fog nodes follow the secure fog procedure in Algorithm 3. Then, fog node i’s exact estimate information $x_{k}^{i}(\;i=1,2,\cdots ,p$) at iteration k cannot be obtained by other neighboring fog nodes through the communication among them.*

**Proof.**  We start by looking at Step 4 of Algorithm 3. Fog node *i* sends its encrypted estimate ${\left[-x_{k-1}^{i}\right]}_{pk_{i}}$ to node *j*. Node *i* will not be able to decrypt it without having node *i*’s private key $sk_{i}$. In Step 5, node *j* encrypts its own estimate using node *i*’s public key $pk_{i}$ and obtains ${\left[x_{k-1}^{j}\right]}_{pk_{i}}$. Perform the addition ${\left[x_{k-1}^{j}\right]}_{pk_{i}}+{\left[-x_{k-1}^{i}\right]}_{pk_{i}}$ and then multiply a private random number $\gamma_{ji}$ to the sum and finally sends it back to node *i*. When node *i* decrypts the message, it obtains $\gamma_{ji}\times \left(x_{k-1}^{j}-x_{k-1}^{i}\right)$ according to the “addition” homomorphic property of the Paillier encryption scheme. Fog node *i* cannot then obtain node *j*’s estimate $x_{k-1}^{j}$ since $\gamma_{ji}$ is a number privately held by node *j* and unknown to node *i*. The same reasoning can be applied to node *j*’s update in Steps 13–15 of Algorithm 3. This completes the proof for Theorem 2. □

**Theorem** **3.** *Assume all edge devices follow the secure edge device procedure in Algorithm 4 and there are n edge devices in fog area i. Then, edge device j’s exact private gradient information $\nabla F_{j}\left(y_{k}^{i}\right)(j=1,2,\cdots ,n$) at iteration k cannot be obtained by other neighboring edge devices through the communication among them.*

**Proof.**  Assume the edge devices are labeled from 1 to *n*, and this is the order that these edge devices perform Step 5 in Algorithm 4. That is, Edge Device 1 will pass its encrypted gradient ${\left[\nabla F_{1}\left(y_{k}^{i}\right)\right]}_{pk_{i}}$ to edge device 2. Edge Device 2 will do summation of the received encrypted gradient from Edge Device 1 with its own encrypted gradient and then pass the sum ${\left[\nabla F_{1}\left(y_{k}^{i}\right)\right]}_{pk_{i}}+{\left[\nabla F_{2}\left(y_{k}^{i}\right)\right]}_{pk_{i}}$ to Edge Device 3. This process will repeat until device *n* obtains the total sum of all the encrypted gradient information $\sum_{j=1}^{n}{\left[\nabla F_{j}\left(y_{k}^{i}\right)\right]}_{pk_{i}}$. In this process, it can be seen that when Edge Device 1 sends its encrypted gradient ${\left[\nabla F_{1}\left(y_{k}^{i}\right)\right]}_{pk_{i}}$ to Edge Device 2, Edge Device 2 cannot infer Edge Device 1’s exact private gradient in plaintext since the gradient is encrypted using fog node *i*’s public key $pk_{i}$ and Edge Device 2 cannot decrypt it without knowing fog node *i*’s private key $sk_{i}$. The same reasoning can be applied into the communication between other edge devices in between. This completes the proof of Theorem 3. □

**Theorem** **4.** *Assume all the fog nodes and edge devices follow the secure procedures in Algorithms 3 and 4, respectively. Suppose there are n edge devices in fog area i. Then, edge device j’s exact private gradient $\nabla F_{j}\left(y_{k}^{i}\right)$ cannot be obtained by fog node i or a curious edge device q over time if the number of edge devices n≥3.*

**Proof.**  First, in Step 6 of Algorithm 4, the last edge device *n* will send the sum of all the encrypted gradients $\sum_{j=1}^{n}{\left[\nabla F_{j}\left(y_{k}^{i}\right)\right]}_{pk_{i}}$ to the corresponding fog node *i*. Fog node *i* will then decrypt this message using its private key $sk_{i}$ but it will only obtain the sum of all the edge devices’ gradients in plaintext $\sum_{j=1}^{n}\nabla F_{j}\left(y_{k}^{i}\right)$ and is not able to pinpoint edge device *j*’s exact gradient $\nabla F_{j}\left(y_{k}^{i}\right)$ if n≥2. Second, we consider the Steps 9 and 11 in Algorithm 3. Edge device *q* is curious about edge device *j*’s private gradient $\nabla F_{j}\left(y_{k}^{i}\right)$. Device *q* can receive $y_{k}^{i}$ from fog node *i* and we further assume that edge device *q* somehow can access fog node *i*’s estimate $x_{k}^{i}$ and learning rate $\eta_{i_{k}}$ over time. Hence, it can access the sum of the gradients $g_{k}^{i}=\sum_{j=1}^{n}\nabla F_{j}\left(y_{k}^{i}\right)$ in plaintext (based on Step 11 in Algorithm 3). If the number of the edge devices n=2, then device *q* can take the difference between $g_{k}^{i}=\nabla F_{j}\left(y_{k}^{i}\right)+\nabla F_{q}\left(y_{k}^{i}\right)$ and its own gradient $\nabla F_{q}\left(y_{k}^{i}\right)$ to obtain device *j*’s gradient $\nabla F_{j}\left(y_{k}^{i}\right)$. When the number of edge devices n≥3, device *q* will not be able to pinpoint the device *j*’s exact gradient $\nabla F_{j}\left(y_{k}^{i}\right)$. This completes the proof for Theorem 4. □

**Remark** **2.** *An important alternative in the literature is Federated Learning (FL) that can be applied to IoT devices to jointly learn a model without sharing their raw data [56]. The limitation is that many FL algorithms are based on the parameter-server architecture that a centralized server exists, and thus it is vulnerable to single point of failure. In our proposed approach, the fog nodes cooperate in a decentralized manner by performing local computation and exchanging information with each other. Regarding privacy and accuracy, differential privacy is adopted by many FL approaches. The foundation of differential privacy is to perturb the query output by adding random noise [35,36]. A fundamental trade-off exists in differential privacy that the accuracy of the solution depends on the level of the noise added. Our approach uses cryptographic-based techniques, and we show that it does affect the accuracy. For latency comparison, it depends on the network environment. Our approach mainly uses local communication and sometimes it can be faster than FL. Similar observations have been made previously [57,58].*

## 5. Experimental Evaluation

**Figure 5:**
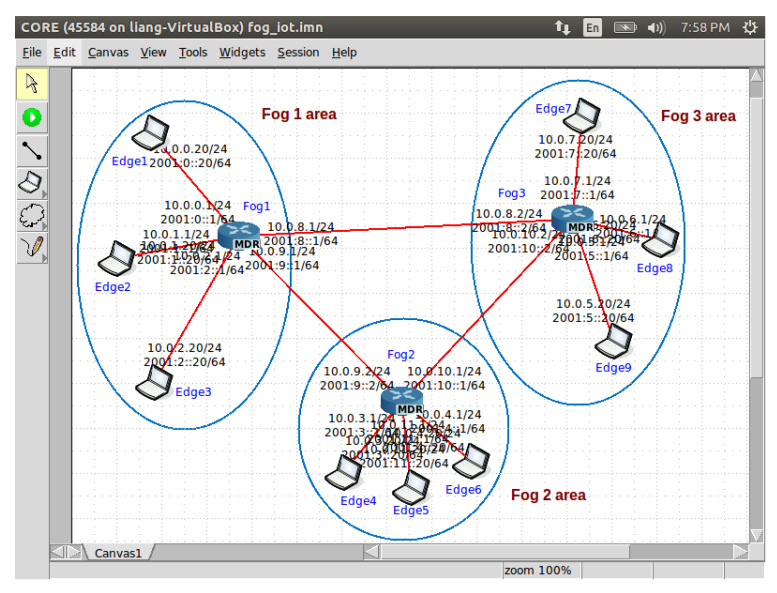
An example of the CORE GUI.

We conducted experiments to evaluate the performance of our proposed distributed algorithms in this section. We investigated two case studies: seismic imaging and diabetes progression prediction. We used Common Open Research Emulator (CORE) [59] to emulate the algorithms performed in fog-enabled IoT systems. An example of the CORE GUI is illustrated in Figure 5. Python Paillier package [60] was used for the implementation of Paillier cryptosystem. We adapted two distributed algorithms in the literature denoted by “Nedic’s method” [33] and “ADL method” [34] to fit the fog empowered IoT system architecture and use them as benchmarks for comparison.

### 5.1. Seismic Imaging

**Figure 6:**
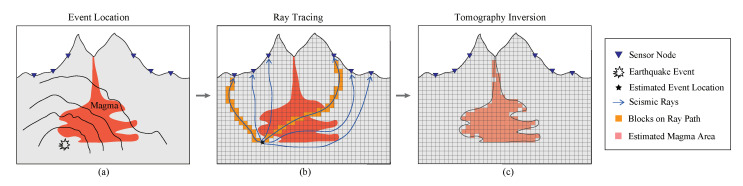
Procedures of seismic imaging. The first step is event localization (**a**), then ray tracing (**b**), and the final step is tomography inversion (**c**). We focus on the last step only in this scenario since it is the main computation stage.

We investigated the performance of our distributed algorithm in the application of seismic imaging. The conventional travel-time based tomography consists of three steps (see Figure 6), and we focused on the last tomography inversion step in this paper. The tomography inversion step aims to obtain the image under the surface using earthquake events and can be modeled as solving a linear system of equations [61]:

(10) Ax=b

where matrix *A* and vector *b* contain the ray and travel-time information. *x* is the unknown vector to be estimated representing the values in blocks. We used AIR tools [62] to generate the data *A* and *b* and also the ground truth *x*.

To fit the formulation in Section 3, we converted (10) into an optimization problem as follows.

(11) $$\min_{x}\;\;\frac{1}{2}{∥Ax-b∥}_{2}^{2}+\lambda^{2}{∥x∥}_{2}^{2},$$

where the first term is for data fitting and the second one is the regularization part. We adopted Tikhonov regularization to help reconstruct the tomography as the measurements in vector *b* is noisy and causing the linear system in (10) to be inconsistent.

Note that this travel-time seismic imaging problem can fit into our the decomposed formulation in Section 3.1 naturally. The local private objective function $F_{i}$ for data generation place *i* is as follows.

(12) $$F_{i}=\frac{1}{2}∥A_{i}x-b_{i}{∥}_{2}^{2}+\lambda_{i}^{2}{∥x∥}_{2}^{2},$$

where the ray and travel-time information in $A_{i}$ and $b_{i}$ are generated in a distributed fashion. The characteristics of the data generated is as follows. The resolution of the tomography is 64×64 and thus the size of *x* is 4096×1. The size of matrix *A* is 16,384×4096 and the size for vector *b* is 16,384×1 accordingly. We emulate a fog-enabled IoT system with 64 edge devices and 8 fog nodes. The data in *A* and *b* are divided evenly for all the edge devices and thus the dimensions for $A_{i}$ and $b_{i}$ are 256×4096 and 256×1, respectively. The regularization parameter $\lambda^{2}$ is set to 1 and $\lambda_{i}^{2}$ is $\frac{1}{64}$ for each edge device *i*. We used fixed momentum β and step size parameter η for all the nodes and all the benchmarks. In each fog area, there are eight edge devices. The connection among fog nodes are randomly generated and each fog node has three neighbors on average. To compare the performance of our proposed algorithm with the benchmarks, two metrics were used as follows.

- **Objective value:** We took the average solution ${\bar{x}}_{k}$ of all the *p* fog nodes and evaluated the objective value of the global function $F\left({\bar{x}}_{k}\right)$. This metric tracks how good of the average model is in reaching optimal over iterations.
  $$F\left({\bar{x}}_{k}\right)=\sum_{i=1}^{m}F_{i}\left({\bar{x}}_{k}\right),\;\mathrm{where}\;\;{\bar{x}}_{k}=\frac{1}{P}\sum_{i=1}^{P}x_{k}^{i}.$$
- **Disagreement:** We took the difference of each fog node’s solution with the average solution. This quantity measures the disagreement among all the fog nodes in their estimates. Hence, it indicates how fast these fog nodes reach consensus.
  $$\sum_{i=1}^{P}{∥x_{k}^{i}-{\bar{x}}_{k}∥}^{2}.$$

**Figure 7:**
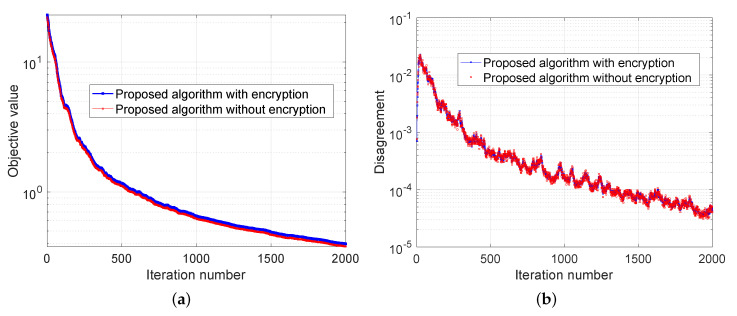
Seismic imaging problem with comparing the accuracy of the proposed distributed algorithm with or without encryption. (**a**) and (**b**) compare the performance of proposed algorithm with and without encryption in terms of objective value and disagreement, respectively.

**Figure 8:**
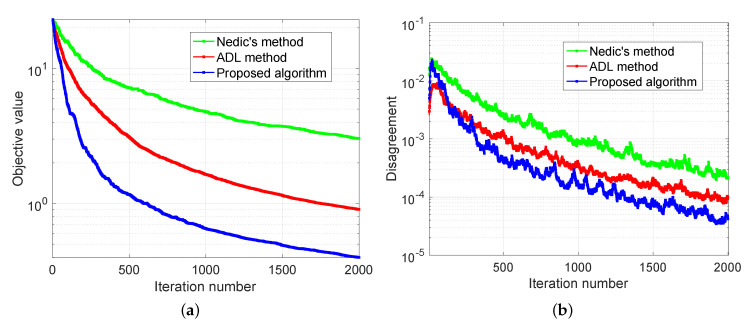
Seismic imaging problem with convergence behavior comparison. (**a**) and (**b**) compare the performance of proposed algorithm with the benchmarks in terms of objective value and disagreement, respectively.

**Figure 9:**
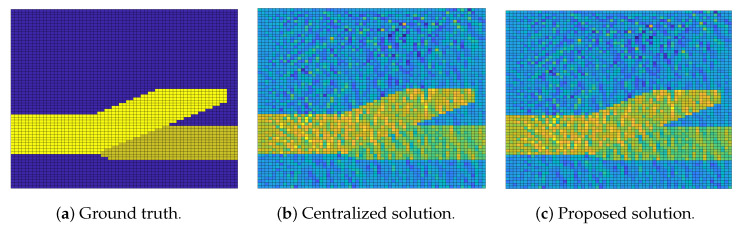
Seismic imaging problem with tomography results comparison. The dimension of the tomography results is 64×64 and hence there are 64 blocks along the vertical and horizontal axes, respectively (**a**–**c**).

The experimental results are demonstrated in Figure 7, Figure 8 and Figure 9. In Figure 7, we compare our distributed algorithm (Algorithms 1 and 2) with the proposed secure protocol (Algorithms 3 and 4) in terms of objective value and disagreement. It can be seen that the encrypted secure protocol is close to the distributed algorithm in model accuracy. It verifies the statement in Theorem 1 and implies that the secure protocol does not affect the accuracy of the solution. Figure 8 shows that our proposed distributed algorithm outperforms the two benchmarks that all the fog nodes can reach the optimal solution and consensus faster in iteration number. In particular, it can be observed that, in Figure 8a, our proposed method can be up to one order of magnitude faster than the benchmarks on reaching the same level of objective value. Finally, we show seismic imaging results in Figure 9. Note that the solution obtained from our distributed algorithm is close to the centralized solution that pre-computed using a centralized solver in advance. This is expected since the goal of our distributed algorithm is to recover the same solution from centralized method. Designing better models (other than (11) using different regularizers and parameters) can possibly produce tomography closer to the ground truth, but it is out of scope of this paper.

### 5.2. Diabetes Progression Prediction

**Figure 10:**
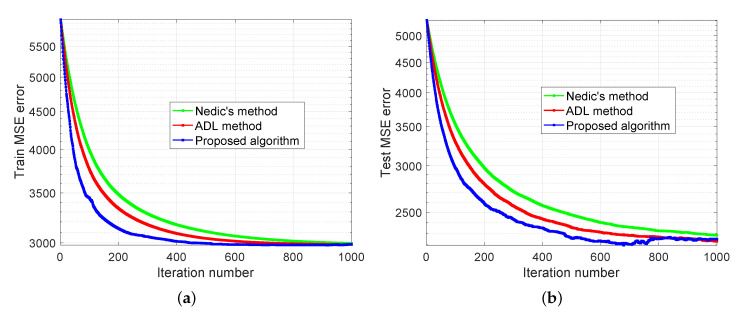
Diabetes progression prediction with 20 edge devices and 5 fog nodes. (**a**) and (**b**) compare the performance of proposed algorithm with the benchmarks in terms of training and testing error, respectively.

**Figure 11:**
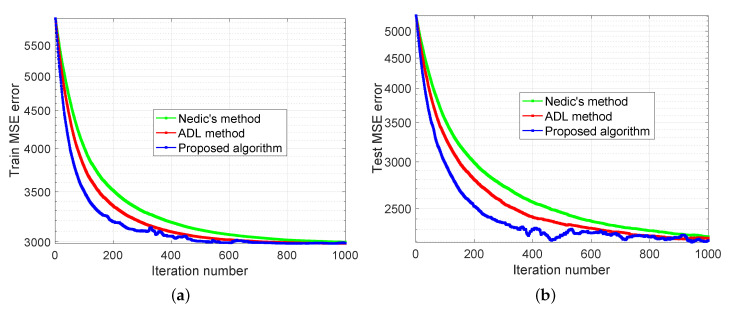
Diabetes progression prediction with 40 edge devices and 10 fog nodes. (**a**) and (**b**) compare the performance of proposed algorithm with the benchmarks in terms of training and testing error, respectively.

We tested our proposed algorithm in a machine learning task for a sensitive medical dataset. The dataset was from sklearn [63]. There are 10 variables: age, gender, body mass index, average blood pressure, and six blood serum measurements. The target is a quantitative measure of the diabetes disease progression. We considered a scenario that multiple hospitals would like to cooperate with each other to obtain a better prediction model equivalent to training on all the data they have. However, the sensitive data maintained by each hospital cannot be moved or leaked during their cooperation and interaction with each other. The problem aforementioned was emulated in a fog-based IoT system as follows. Each hospital maintains an edge device containing the patient data. There are 442 instances in the dataset and 50 records were used as test set and the remaining were divided into the edge devices evenly (approximately) as their local private training data. We considered two cases: (1) There are 20 edge devices and 5 fog nodes. The connections among fog nodes are randomly generated and each fog has two neighbors. (2) There are 40 edge devices and 10 fog nodes. The connections among fog nodes are randomly generated and each fog has five neighbors on average. A linear regression model was adopted for this problem and mean square error was used for measuring the training and testing error. The learning rate η and the momentum parameter β were set to 1.0 and 0.5 in all cases, respectively. The results are illustrated in Figure 10 and Figure 11, and it can be observed that our proposed distributed algorithm is consistently superior in terms of training and testing error in both cases. For instance, in Figure 10a, we can see that, for the level of training MSE error to decrease to 3120, our proposed needs 200 iterations while ADL and Nedic’s methods require around 320 and 420 iterations, respectively. It implies that our approach can be up to two times faster in reaching a reasonable accuracy. Notice that the performance gaps between our proposed algorithm and the benchmarks are not as significant as shown in the seismic imaging case study (Section 5.1). One possible reason is that the diabetes progression prediction problem is “simple” comparing to the seismic imaging problem due to the differences between their dimension of decision variables and number of instances. As a result, it would be relatively easier for all the methods to optimize the parameters to fit the data and thus the performance gap might experience a shrink in this scenario.

### 5.3. Enron Spam Email Classification

We studied the spam email classification problem in a fog-computing environment. The Enron dataset was used to train logistic regression models to classify email as spam or ham. The dataset contains data from about 150 people from senior management of Enron, organized into folders [64]. In total, 5000 emails from Enron Folder 1 were used for training. Among them, 26% are spam and 74% are ham emails. We pre-processed the emails and kept only frequent words. The dimension for the features is 7997 determined by counting the frequent words. The training error (fraction that the training model is wrong in classification) and training log loss (value of the loss function) were used to measure the learning process. Fog nodes were considered to form a mesh network such that there is a direct link between any pair of fog nodes. The learning rate η and the momentum parameter β were fixed to 1.0 and 0.5, respectively.

**Figure 12:**
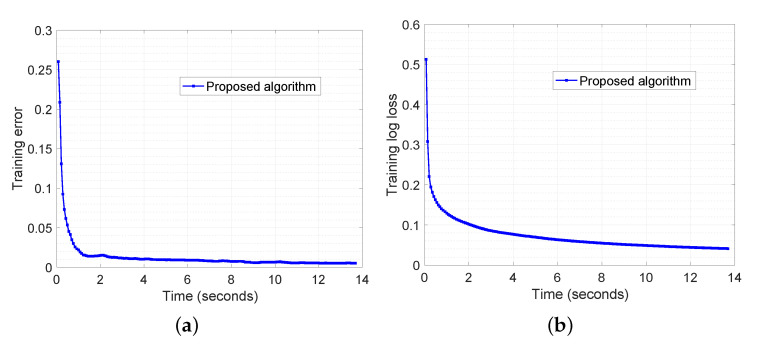
Enron Spam Email Classification with 100 edge devices and five fog nodes. The average model obtained from five fog nodes is illustrated. The latency for communication is set to 5 ms, and bandwidth is set to 10 mbps. (**a**) and (**b**) depict the performance of proposed algorithm in terms of training error and training log loss, respectively.

**Figure 13:**
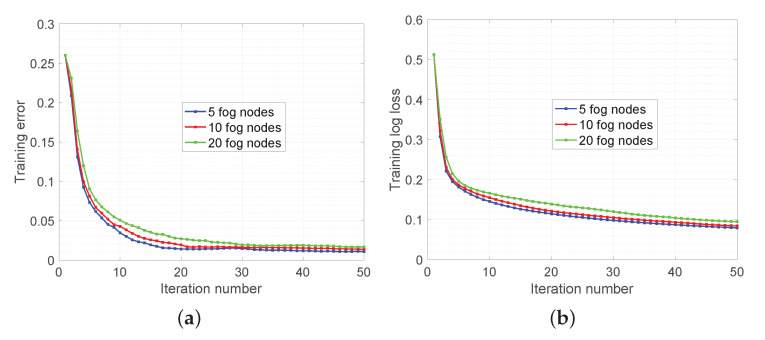
Enron Spam Email Classification with various numbers of fog nodes: 5, 10, and 20. (**a**) and (**b**) illustrate the performance of proposed algorithm in terms of training error and training log loss, respectively.

The experimental results are demonstrated in Figure 12 and Figure 13. In Figure 12, we show the training error and log loss along the wall-clock time. We tested with 100 edge devices and they were evenly divided into five fog areas. The training dataset was split into edge devices evenly such that each device contained 50 emails. The latency of communication was set to 5 ms and the bandwidth was set to 10 mbps. It can be observed that the training error is below 0.05 (accuracy is above 0.95) after around 1 s (Figure 12a). The training log loss (Figure 12b) keeps decreasing and it indicates that the logistic regression model is still updating. In Figure 13, we test the proposed algorithm with various number of fog nodes: 5, 10, and 20. It can be seen that the training model is slower reaching the same accuracy as the number of fog nodes increases. This observation is expected because each fog area will hold fewer data samples if more fog nodes are used and it takes more iterations for each fog node to reach the same accuracy.

## 6. Conclusions and Future Directions

This papers presents a new distributed algorithm for data analytics in fog-enabled IoT systems. The edge devices transmit gradient information into their corresponding fog nodes. Fog nodes cooperate with each other in a decentralized fashion to obtain the global solution by exchanging their estimates. To protect the privacy of edge devices (raw data holders), a privacy-preserving protocol has been invented combining the proposed distributed algorithm with Paillier homomorphic encryption. The secure protocol guarantees that any edge device’s private information about its raw data would not be leaked during the computation and analytics processes. To evaluate the effectiveness of our proposed approach, we conducted empirical studies on three applications. On seismic imaging, our proposed algorithm shows an up to one order of magnitude acceleration in minimizing the objective value. On diabetes progression prediction problem, our method can be up to two times faster in terms of reaching a reasonable training MSE error. On Enron spam email classification task, we show that the proposed algorithm can achieve above 0.95 accuracy in 1 s wall-clock time with 5 ms communication latency and 10 mbps bandwidth network configuration. For future work, we plan to investigate the effectiveness of our proposed idea in deep learning models.

## Abbreviations

The following notations and abbreviations are used in this manuscript:

IoT	Internet of Things
$x_{k}^{i}$, $y_{k}^{i}$	Fog node *i*’s estimate and auxiliary variable at iteration *k*
$\beta_{k}^{i}$, $\eta_{k}^{i}$	Fog node *i*’s momentum parameter and step size parameter at iteration *k*
$pk_{i}$, $sk_{i}$	Fog node *i*’s public and private key
$\gamma_{ij}$	Fog node *i*’s private random number prepared for node *j*
$\gamma_{ji}$	Fog node *j*’s private random number prepared for node *i*
